# Restoring Wnt signaling in a hormone-simulated postpartum depression model remediated imbalanced neurotransmission and depressive-like behaviors

**DOI:** 10.1186/s10020-023-00697-4

**Published:** 2023-07-25

**Authors:** Binglu Ye, Yawei Yuan, Rui Liu, Haitao Zhou, Yujie Li, Zhihao Sheng, Tianyu Li, Bing Zhang, Zhendong Xu, Yang Li, Zhiqiang Liu

**Affiliations:** 1 Department of Anesthesiology, Shanghai First Maternity and Infant Hospital, School of Medicine, Tongji University, Shanghai, 201204 China; 2 Shanghai Key Laboratory of Maternal Fetal Medicine, Shanghai Institute of Maternal-Fetal Medicine and Gynecologic Oncology, Clinical and Translational Research Center, Shanghai First Maternity and Infant Hospital, School of Medicine, Tongji University, Shanghai, 201204 China; 3 State Key Laboratory of Drug Research and Key Laboratory of Receptor Research, Shanghai Institute of Materia Medica, Chinese Academy of Sciences, Shanghai, 201203 China; 4 University of Chinese Academy of Sciences, No.9A Yuquan Road, Beijing, 100049 China; 5 National Clinical Research Center for Aging and Medicine, Huashan Hospital, Fudan University, Shanghai, 200040 China

**Keywords:** Postpartum depression (PPD), Neurotransmission, Hippocampus, CA1, Wnt signaling

## Abstract

**Background:**

Postpartum depression (PPD) is a prevalent mental disorder that negatively impacts mothers and infants. The mechanisms of vulnerability to affective illness in the postpartum period remain largely unknown. Drastic fluctuations in reproductive hormones during the perinatal period generally account for triggering PPD. However, the molecular mechanism underlying the PPD-like behaviors induced by the fluctuations in hormones has rarely been reported.

**Methods:**

We utilized hormones-simulated pseudopregnancy (HSP) and hormones-simulated postpartum period (HSPP) rat models to determine how drastic fluctuations in hormone levels affect adult neurotransmission and contribute to depressive-like behaviors. The electrophysiological response of CA1 pyramidal neurons was evaluated by whole-cell patch clamping to identify the hormone-induced modulations of neurotransmission. The statistical significance of differences was assessed with One-way ANOVA and t-test (p < 0.05 was considered significant).

**Results:**

Reproductive hormones withdrawal induced depressive-like behaviors and disturbed the balance of excitatory and inhibitory transmission in the pyramidal neurons in the hippocampus. Molecular analyses revealed that the blunted Wnt signaling might be responsible for the deficits of synaptic transmission and behaviors. Activation of Wnt signaling increased excitatory and inhibitory synaptic transmission in the hippocampus. Reactivation of Wnt signaling alleviated the anhedonic behaviors and abnormal synaptic transmission.

**Conclusions:**

Restoring Wnt signaling in the hormones-simulated postpartum period rat models remediated depression-related anhedonia symptoms and rebalanced the excitation/inhibition ratio by collectively enhancing the plasticity of GABAergic and glutamatergic synapses. The investigations carried out in this research might provide an alternative and prospective treatment strategy for PPD.

**Supplementary Information:**

The online version contains supplementary material available at 10.1186/s10020-023-00697-4.

## Background

Postpartum depression (PPD) has been identified as a concern because it may impact both maternal and newborn health, and PPD is a major maternal health issue worldwide (Stewart and Vigod 2016; Premji et al. 2021). Maternal suicide related to PPD has soared to be the second leading cause of death in postpartum women (Yim and Dunkel Schetter 2019). However, less than 20% of mothers with PPD receive treatment or are diagnosed, as the underlying mechanisms are unclear and there is a lack of effective therapies. Fluctuations in ovarian hormone levels are thought to be one of the triggers of postpartum depression. Oestrogen (E2) and progesterone (P), for instance, are altered more than 100 times during the perinatal period. Therefore, hormone replacement therapy has been studied as a treatment previously (Stewart and Vigod 2016). However, hormonal interventions are accompanied by several serious, severe side effects, including coronary heart disease, stroke, and breast cancer (Rozenberg et al. 2013). The molecular mechanisms of the hormonal fluctuations that cause PPD are still largely unknown. Therefore, it is crucial to investigate how hormonal fluctuations cause PPD symptoms and to seek less risky and more effective therapeutic approaches.

Notably, mental illness does not worsen or develop during pregnancy, and adverse affective symptoms such as anhedonia, self-harm, suicide, and psychiatric admission even decrease during gestation (Langan Martin et al. 2016). Nevertheless, psychiatric symptoms tend to become more severe after delivery among women who have a history of mental illness (Cantwell 2021; Schnakenberg et al. 2021). However, the fundamental mechanisms of these symptoms are largely unknown. Therefore, in this study, we utilized hormones-simulated pseudopregnancy (HSP) and hormones-simulated postpartum period (HSPP) rat models, which have been described previously (Suda et al. 2008; Stoffel and Craft 2004). We aimed to identify the fundamentally different mechanisms of these two perinatal stages in this study, which might contribute to a better understanding of the pathogenesis of PPD and provide us with novel treatment options in the future.

A disturbance of the excitation/inhibition (E/I) ratio in the cortex may lead to aberrant functional connectivity and altered synaptic levels of excitatory and inhibitory neurotransmitters, thus playing a key role in anxiety and depression(Pham and Gardier 2019; Hartmann et al. 2017; Lener et al. 2017). Glutamatergic excitatory pyramidal neurons and GABAergic inhibitory interneurons constitute a complex neuronal circuit in the cortex and modulate the E/I balance. Glutamate receptors, especially AMPA (α-amino-3-hydroxy-5-methyl-4-isoxazolepropionic acid) receptors (AMPARs) play a key role in neurotransmission, neurogenesis, and neuron plasticity, as well as memory and emotion regulation (Ferguson and Gao 2018). GABA_A_ receptors (GABA_A_Rs, γ-Aminobutyric acid sub-type A receptors) play a critical role in modulating rapid inhibitory neurotransmission in the central nervous system (Jacob et al. 2008). Decreased GABAergic plasticity has been implicated in PPD pathogenesis in previous research (Maguire 2008). Nevertheless, it is still unclear whether hormone withdrawal impacts glutamatergic excitatory and GABAergic inhibitory neurotransmission and causes a disturbance of the E/I balance, and we investigated this issue in this research.

Wnt signaling is a principal pathway of adult neurogenesis in the hippocampus (Lie et al. 2005; McLeod et al. 2018; Gao et al. 2021). Hippocampal Wnt signaling goes awry in various neuropsychiatric and neurodegenerative diseases, such as depression disorders, Alzheimer’s disease, and Down syndrome (Roy et al. 2020; Contestabile et al. 2013). During the perinatal period, the neurogenesis of the maternal brain is one of the important processes of neural plasticity modifications that underlie the aetiology of mental disorders such as postpartum depression (Leuner 2016; Slattery and Hillerer 2016; Guzman et al. 2018; Hillerer et al. 2014). There have been reports of interactions between ovarian hormones and Wnt signaling (Fortress and Frick 2016). For example, oestrogen protects the hippocampus from cerebral ischaemia by upregulating Wnt signaling (Scott and Brann 2013). However, few studies have focused on whether hormone withdrawal could induce Wnt signaling dysfunction and be implicated in the development of PPD. In this research, we examined whether Wnt signaling modulates the plasticity of glutamatergic excitatory and GABAergic inhibitory synapses and the E/I balance in the hippocampus and thus contributes to depressive-like behaviors in the HSPP rat model.

This investigation based on hormone-simulated perinatal rat models showed that dysfunctional Wnt signaling in the hormones-simulated postpartum period (HSPP) group may be related to the delay in the return of functional GABA_A_Rs to baseline levels and may influence the plasticity of synapses following hormone withdrawal. Restoring Wnt signaling in the HSPP rat contributed to alleviated depressive-like behaviors, which was achieved by enhancing the plasticity of inhibitory and excitatory synapses in the hippocampal region and rebalancing the excitation/inhibition ratio. We hope these findings could provide a potential target and alternative treatment for PPD. We hope it will help more women suffering from PPD to be cured at an early stage, lessening the burdens on the families of affected mothers and benefiting women’s health care in society.

## Materials and methods

The animal studies and experimental procedures were approved by the Animal Care Committees of the Shanghai Institute of Materia Medica, Chinese Academy of Sciences.

### Animals

Female Sprague Dawley rats (Beijing Vital River Laboratory Animal Technology Co., Ltd.), weighing 30-45 g (15-20 days of age) and 240-260 g (9-10 weeks of age) were used in this study. All rats were housed (three in one cage) under constant environmental conditions (temperature 23 ± 2°C, humidity 55 ± 5%) and maintained on a 12-hour/12-hour light/dark cycle (7:00 AM–7:00 PM lights on). All rats were raised with food and water ad libitum before and after all procedures.

### Hormone-simulated pseudopregnancy (HSP) and hormones-simulated postpartum period (HSPP) rat models

**Fig. 1:**
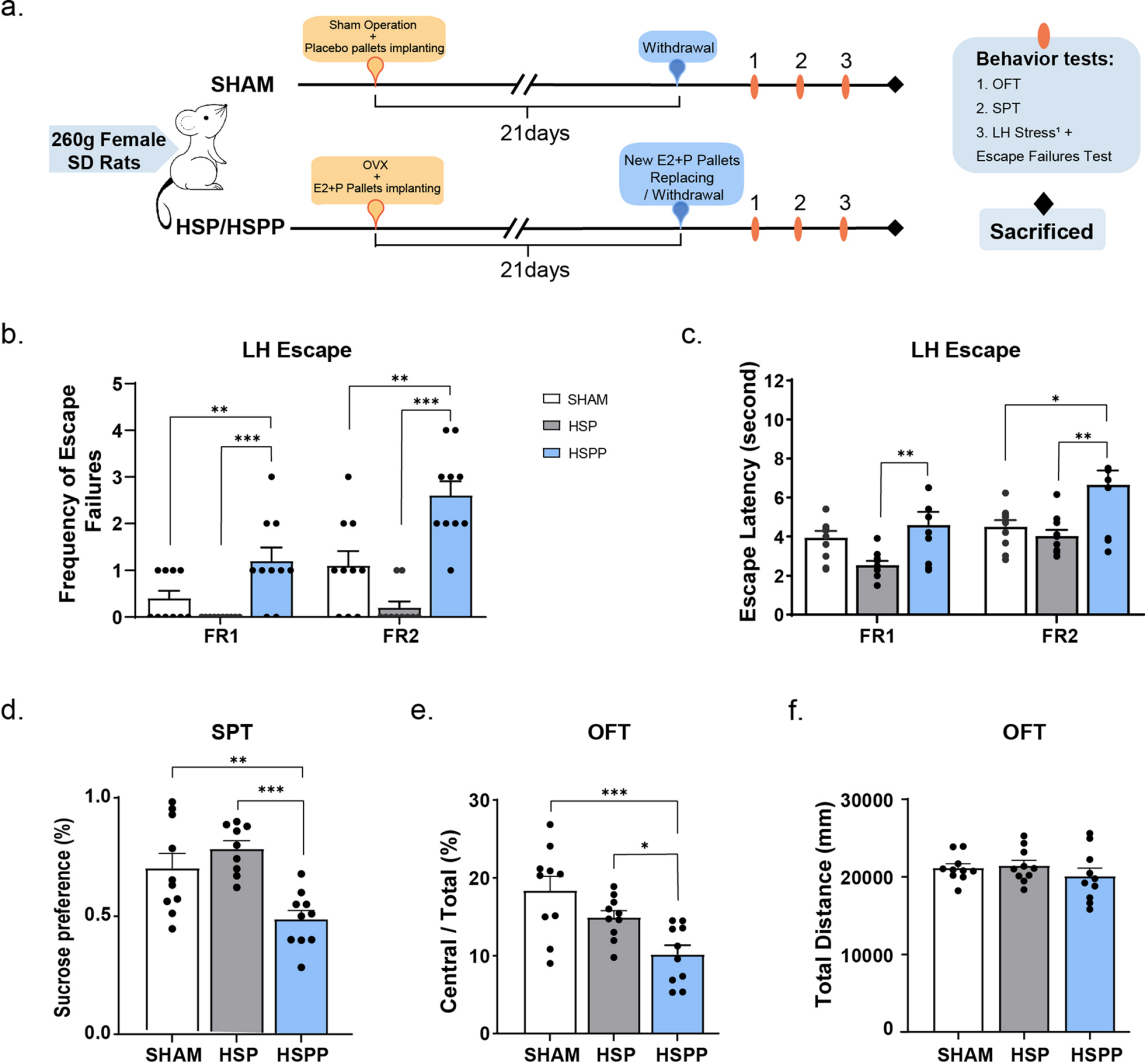
Hormone withdrawal induced depressive-like behaviours.** a** The schedule for building the HSP and HSPP model and the arrangement behaviour tests. The Sham operation (SHAM in below) group was set as control. All behaviour tests were procedure after hormone-withdrawal/sham hormone-withdrawal. (SHAM, n=10; HSP, n=10; HSPP, n=10) **b**, **c** Summary of the three model groups' escape failures and escape latency in both the ^2^FR1 and FR2 paradigms. **d** The sucrose preference test (SPT) was measured for two hours in the three model groups (SHAM, n=10; HSP, n=9; HSPP, n=10). (There was one rat from the HSP group who barely not consumed liquid, so deleted one rat from that group.) **e** The open field test (OFT), and the total moved distance of model animals. **f** Open field test (OFT), the percentage of central distance/total distance of three groups was evaluated. All data were assessed with One-way ANOVA. Turkey’s multiple comparisons test was performed in comparison of the SHAM *versus* the HSP group, the HSP *versus* the HSPP group and the SHAM *versus* the HSPP group. There were no significant differences between the SHAM and the HSP group in all comparisons. Only significant comparisons were displayed in the figures. Data represent means ± SEM. *p<0.05, **p<0.005, ***p<0.001. ^1^LH-stress: learned-helpless stress (inescapable electric foot shock)**.** ^2^FR: fixed-ratio, FR1 (one cross to terminate the shock) and FR2 (two crosses to terminate the shock)

We referenced the procedures in previous research (Suda et al. 2008) to establish the HSP and HSPP models. Adult female Sprague Dawley rats weighing 240–260 g were used in this model. The rats of both the HSP and the HSPP groups were bilaterally ovariectomized (OVX) under pentobarbital/atropine anaesthesia. The sham operation group (SHAM in below) underwent the same operating procedures as the HSP and HSPP groups, in which the equally weighing adipose tissue adjacent to the ovaries was resected. The sham operation group was set as a control group and paralleled virgin rats (without reproductive experiences). Details about the procedures and hormones of each group are shown in Table 1**.** The schedule of establishing this animal model and behaviors testing arrangement is shown in Fig. 1a All rats were implanted with continuous release pellets (21-day release of 0.5 mg of 17ß oestradiol [E2] and 50 mg of progesterone [P4] for the HSP and HSPP groups or placebo for the SHAM group) (*Innovative Research of America, Sarasota, Florida*) to mimic serum hormone levels during human pregnancy. The 21 days with pellets implanted was defined as the simulated gestation period (GD). On the 21st day after the OVX procedures, all rats from the three different groups were anaesthetized again to undergo a hormone withdrawal operation, in which the previously implanted pellets were removed. The HSP group was implanted with new hormone-release pellets at the same time to maintain the same hormone level as during the gestation period. The stage after the hormone withdrawal operation was regarded as the postpartum period (PD), and the behaviors tests and other experiments, such as neuroelectric physiological tests, were all performed during this time.

**Table1:** Groups of experiments

Group	OVX	E2 + P Pallets Implanting	Hormones WD
SHAM	Sham Operation	Placebo	Sham WD Procedure
HSP	+	+	− (New pallets replacement)
HSPP	+	+	+

E2 + P Pallets: 0.5 mg of 17ß estradiol [E2] and 50 mg of progesterone 21-day release pallets

*WD* Withdraw

### Behaviors tests

#### Open field test (OFT)

The open field test (OFT) is a standard method to assess spontaneous locomotor activity. The rats were comforted and placed in the central of a square arena (size: 100 × 100 × 45 cm) that was made of black acrylic plastic. Rats were allowed to explore the box free for 10 min during the dark cycle (7:00 PM-8:00 PM) and were recorded with video on PD2 (two days after hormone withdrawal). The moving trail of each animal was calculated later.

#### Escape failure test after learned helplessness stress

We subjected rats to learned-helpless stress in the postpartum period to mimic the stressful events that occur in the human puerperium. The frequency of escape failures was tested on the next day to measure the helplessness of rats after acute stress. The mean escape failures and the mean escape latencies for each group reflected the hopelessness symptoms of the animals. Hopelessness is a major symptom of depression(Schramm et al. 2020). The procedure of this experiment was described in previous articles(Suda et al. 2008; Baka et al. 2017). Rats were subjected to learned-helpless stress on PD4 (4 days after hormone withdrawal) during the light cycle (11:00 AM-3:00 PM). Rats were placed in separate shuttle boxes with inescapable electric foot shocks. Every rat was subjected to 60 inescapable shocks (0.7 mA intensity; 15-s duration; at random intervals but mean 30 s; range 22 to 38 s). At same time on PD5 (24 h later), all rats were placed back into the shuttle box, and shuttle escape learning was tested in two paradigms. We used fixed-ratio (FR) trials with FR1 (one cross to terminate the shock) and FR2 (two crosses to terminate the shock). Every rat was first subjected 5 FR1 tests followed by 25 FR2 tests. The frequency of escape failures for the FR1 and FR2 trials of each group was analysed separately, and the mean latencies to terminate the electric shock were calculated after the experiments. (Sham-operation, n = 10; HSP, n = 10; HSPP, n = 10).

#### Sucrose preference test (SPT)

The sucrose preference test (SPT) is the method that best assesses anhedonia in rodents. This standard test procedure (Liu et al. 2018) was performed on the first experimental day (GD1). All rats were provided with two bottles of regular water to adapt to getting water from both sides. Rats were separately single-caged on Day 21 (GD21) after the hormone-withdrawal (or sham withdrawal) operation but still given two bottles water to choose from exclude the possibility of location preference. Sucrose preference was tested on PD3 (Day 24) during the light cycle (9:00 AM-11:00 AM). All animals were deprived of water for 12 h the day before (from PD2 at 9:00 PM to PD3 at 9:00 AM). A bottle of 1% sucrose solution and a bottle of regular water were prepared for every rat before the test, and the weight of each bottle was recorded. The sucrose solution and water bottles were gently placed in the cage to ensure that they did not leak. One hour later, both bottles were removed, weighed again, and placed back in the cage with their position interchanged. The bottles were weighed and recorded again another hour later. Sucrose preference was calculated using the following formula: *sucrose preference* = *(sucrose solution intake/total solution intake)* × *100%.*

The total solution intake is the sum of the sucrose solution consumed and the regular water consumed. Normal rats were satisfied with sweets so their sucrose preference ratio was usually greater than 0.5. No sucrose preference, i.e., anhedonia, was defined when the ratio of sucrose preference was approximately 0.5 or less.

#### Body weight measurement

Body weights was measured during the light cycle before that day’s behaviors tests were performed. The first measurement was taken on GD0, meaning the day before the pregnancy period, on which day rats were bilaterally ovariectomized (or sham-operated) and then implanted with 21-day hormone-release pallets. The other five times body weight was measured during the gestation period were GD4 (Day 4 of the gestation period), GD8, GD12, GD16, and GD21. GD21 was the last day of gestation of rats, and the rats were weighed before the hormone-withdrawal (or sham withdrawal) operation. During the postpartum period, body weight was measured on PD2 (Day 2 of the postpartum period), PD4, and PD6 (Additional file 1**: **Fig. S1a). Moreover, we calculated the gain in the body weight of each group on each test day (Additional file 1**: **Fig. S1b).

#### Administration of an agonist of Wnt signaling

The agonist of Wnt signaling used in this study was Wnt agonist 1 (AMBMP hydrochloride, 2-Amino-4-[3,4-(methylenedioxy)benzylamino]-6-(3-methoxyphenyl)pyrimidine, s8178, Selleck), which activates Wnt signaling dependent on beta-catenin- and TCF (transcription factor)-transcriptional activity. AMBMP was dissolved in DMSO to 150 mM for storage. The working concentration of AMBMP was 1 mM diluted with normal saline (NS). Each animal was intraperitoneally injected (i.p.) with 5 mg/kg BW for a single dose. Rats in the control group were injected with the same volume of a solution with a DMSO/NS proportion (1:149) equal to that of the AMBMP working solution.

To determine the effective administration regimen, two schemes were devised to test the effect of activating Wnt signaling by AMBMP on GABA_A_Rs functions. The brains of rats were sampled separately after one hour and three days of continuous administration. Female SD rats weighing 30-50 g (2–3 weeks aged) were used in this experiment. Control groups of each scheme were injected equal volume of DMSO/NS mixed solution. The IPSCs and EPSCs were recorded on CA1 pyramidal neurons on one side of hippocampal brain slices, and target protein expression was examined on the other side after administration of each rat.

#### Western blots

Animals were anaesthetized and decapitated on PD6 (Day 6 of the simulated postpartum period). The hippocampus tissues of rats were dissected immediately and homogenized in ice-cold RIPA buffer (1% NP-40, 1% sodium deoxycholate, 0.1% SDS, 150 mM NaCl, 50 mM Tris–HCl, pH 7.8, 1 mM EDTA) (Biyuntian, Shanghai). Membrane protein of hippocampal tissues was isolated by Mem-PER Plus Membrane Protein Extraction Kit (89,842, Thermo Scientific). All the samples were analysed by 10% sodium dodecyl sulfate–polyacrylamide gel electrophoresis, then transferred to 0.25 μm polyvinylidene difluoride membranes. The primary and secondary antibodies used to probe were: rabbit anti-β-tubulin (1:1000, Cell Signaling Technology), rabbit anti-PSD95 (1:1000, Cell Signaling Technology), rabbit anti-pan-cadherin (1:1000, Abcam), rabbit anti-sFRP1 (1:1000, Abcam), rabbit anti-γ2-GABA_A_R (1:1000, Cell Signaling Technology), mouse anti-δ-GABA_A_R (1:1000, Cell Signaling Technology), mouse anti-α5-GABA_A_R (1:1000, Cell Signaling Technology), rabbit anti-β-catenin (1:1000, Cell Signaling Technology), rabbit anti-Glutamate Receptor 1 (AMPA subtype) (1:1000, Cell Signaling Technology), and Goat anti-rabbit IgG (1:5000, Abcam), Goat anti-mouse IgG (1:5000, Abcam). Image Processing and Analysis in Java (ImageJ) software were used to measure the density of each band on Western blots. The relative expression level of each target protein was calculated as the ratio of target protein band density to β-Tubulin density.

#### Transcriptomic sequencing

Animals for transcriptomic sequencing were naïve to all behaviors tests. Rats from the HSP and HSPP groups were decapitated after being anaesthetized on PD6. All hippocampi were dissected carefully with RNA enzyme-free instruments, placed on ice and immediately frozen in liquid nitrogen. Before HiSeq 2000 sequencing, messenger RNA (mRNA) was enriched by OligoT and random hexamer-primed cDNA synthesis, and a second PCR was performed. Then the data were analysed by Shanghai Personalbio Technology (Shanghai, China).

### Brain slice preparation and electrophysiological analysis

#### Brain slice preparation

After being anaesthetized, rats underwent heart perfusion with artificial cerebrospinal fluid (aCSF). Young rats weighing 30–45 g (15–20 days of age) and adult rats weighing 240–260 g (9–10 weeks of age) were perfused with cold (~ 4 °C) and oxygen-saturated aCSF (Additional file 2**: Table S**1-S3). After being decapitated, the brain was removed and submerged in cold (~ 4℃) aCSF with carbogen (95% O_2_, 5% CO_2_) to prepare for sectioning. Hippocampal slices (300–320 μm thick) were cut with a vibratome (Leica VT1000s, USA) in the sagittal plane. Brain slices were quickly and gently placed into an incubated chamber and submerged in carbogen (95% O_2_, 5% CO_2_)-saturated aCSF (~ 29 °C) for at least 1 h.

The components of the aCSF were adjusted based on the ages of the rats to ensure that the neurons were in a good condition for patch clamping and postsynaptic currents recording. For younger rats, normal aCSF was used for perfusion, incubation, and continuous pumping in the recording stage, while modified aCSF was used during the slicing stage. HEPES-aCSF (with 3 mM NAC, N-acetyl-L-cysteine, *Aladdin*) was used for perfusion and incubation in adults. For slicing, NMDG-aCSF (with 3 mM NAC) was used., Adult brain slices were bathed in HEPES-aCSF during the recording stage. All aCSFs were adjusted to the same pH range (7.3–7.4) and to 305–310 mOsm. The aCSF formulas used were those from an early publication (Liu 2016) and modified by our group.

#### Electrophysiological analysis

After incubation, brain slices underwent recording in a recording chamber with continuously pumped and carbogen-bubbled (95% O2, 5% CO2) aCSF (3.5 ml/min) at room temperature (~25°C). CA1 pyramidal neurons could be identified by their morphological features under the optical microscope (Olympus, Japan). Single neuron recordings were made in the whole-cell configuration connected to Multiclamp 700B amplifier (Molecular Devices, USA) in Gap-free mode. All pipettes were fire-polished to a resistance range of 4-5 MΩ and filled with intracellular solutions. Neurons were voltage clamped at -80 mV and -60 mV (holding potential) separately for the recording of their inhibitory postsynaptic current (IPSC) and excitatory postsynaptic current (EPSC). The IPSC intracellular solution contained (in mM): 110 CsCl, 30 K-Gluconic, 0.1 CaCl2, 10 HEPES, and 4 Mg-ATP, 0.3 GTP, 1.1 EGTA, adjusted pH to 7.3 with CsOH. The EPSC intracellular solution contained (in mM): 115 CsMeSO3, 20 CsCl, 10 HEPES, 2.5 MgCl2, 4 Na2-ATP, 0.4 Na-GTP, 10 Na-phosphocreatine, and 0.6 EGTA, adjusted pH to 7.35with CsOH. CNQX (20 μM) (Apexbio) and MK801 (10 μM) (MCE) were added to the pumped aCSF media to inhibit excitatory -amino-3-hydroxy-5-methyl-4-isoxazolepropionic acid receptor (AMPA) and N-methyl D-aspartate receptor (NMDAR) currents before recording spontaneous-IPSC (sIPSC). All voltage-clamped data were filtered at 2.0 kHz and sampled at 20.0 kHz with Digidata 1440A (Molecular Devices, USA). The sIPSC and spontaneous-EPSC (sEPSC) data were analysed offline with analysis software (Minianlysis, Synaptosoft).

#### Statistical analyses

We used the statistical software GraphPad Prism9.0.0 (GraphPad Software Inc.) to do statistical analysis for this research. Values were shown as individual samples and or mean ± SEM. The statistical significance of differences was assessed with One-way ANOVA and t-test (p<0.05 was considered significant).

## Results

### Hormone withdrawal induced depressive-like behaviors

Shiro Suda and colleagues developed hormone-simulated pseudopregnancy (HSP) and hormones-simulated postpartum period (HSPP) rat models in previous research to attempt to imitate the phenotype of PPD (Suda et al. 2008). Our research observed similar depressive-like behaviors in the HSPP group animals with hormone-withdrawal treatment in the simulated postpartum period. In both the FR1 (one cross to terminate the shock) and FR2 (two crosses to terminate the shock) paradigms, rats in the HSPP group displayed significantly more escape failures (Fig. 1b), and showed similar tendency in mean escape latencies, and the HSPP group took significantly longer to terminate electric shock in every escape than the other two groups (Fig. 1c). In the HSP group, animals scarcely had failures to escape and escaped quickly in every shock compared with the other two groups (Fig. 1b, c). In the sucrose preference test, the HSPP group showed a significant reduction in preference for sucrose on the third day of the simulated postpartum period (Fig.1d). These results indicated that hormone-withdrawal induced the hopelessness and anhedonic symptoms in rats in the HSPP group.

Moreover, the percentage of central distance/total distance significantly decreased in the HSPP group, indicating the anxious symptoms of the rats (Fig. 1e), which coordinated with the clinical observations (Vigod et al. 2016). Rats from three different groups displayed no differences in the total moving distance in the open field test (Fig.1f), meaning that the locomotor activity of rats was not influenced by hormone withdrawal or operation procedures. That is, the HSPP group took much more time to escape than the other two groups, not because of motor dysfunction but as a consequence of a despairing mood that might have been induced by hormone withdrawal. The body weight of animals was also influenced by fluctuations in hormones (Additional file 1: Fig. S1). Collectively, these observations show that we established a reliable PPD-like rat model through hormone-withdrawal procedures.

### Hormone-withdrawal disturbed the excitatory/inhibitory balance in the hippocampus

**Fig. 2:**
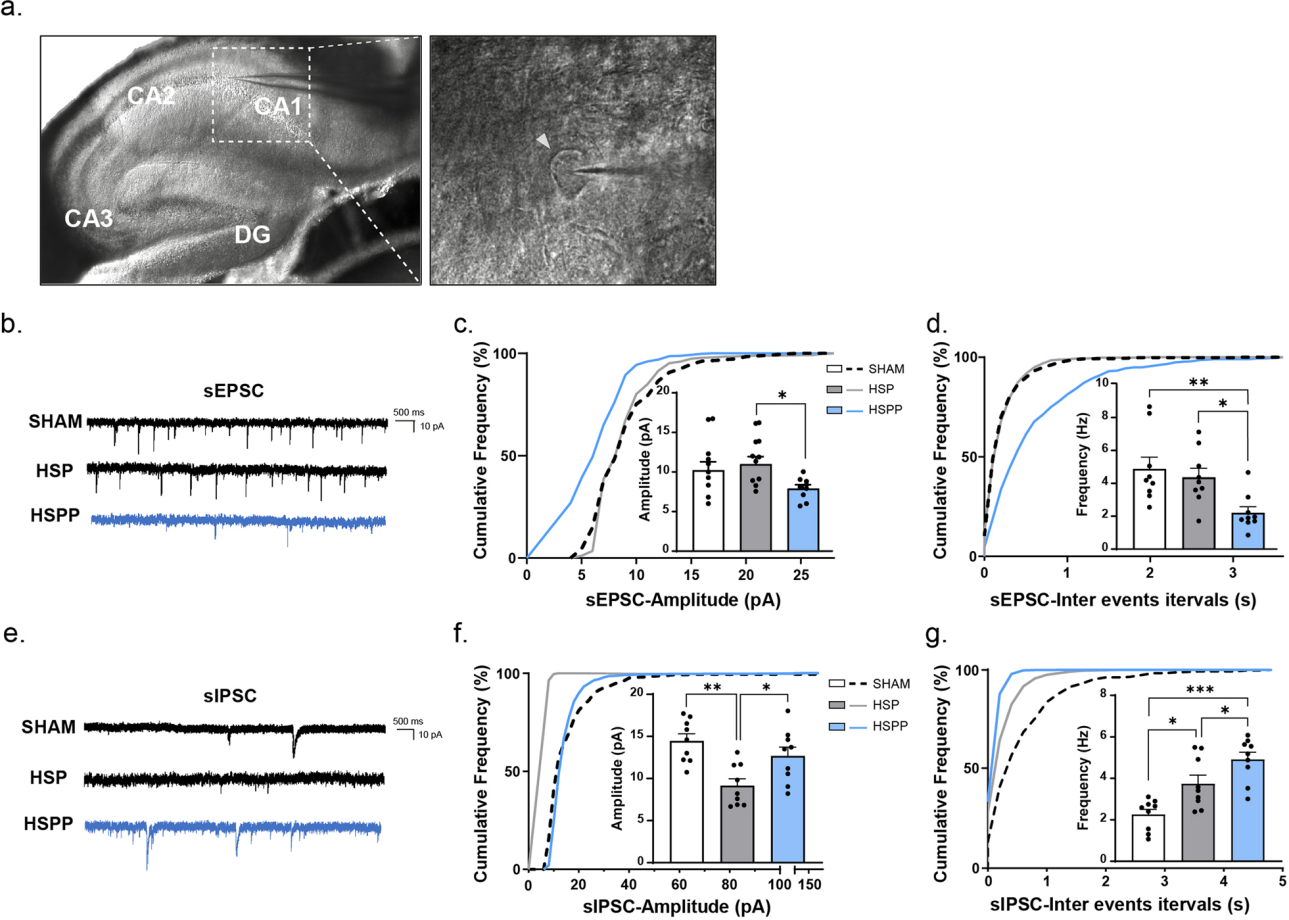
Hormones withdrawal disturbed the excitatory/inhibitory ratio of pyramidal neurons from CA1 in the hippocampus. **a** Schematic diagram of glass microelectrode recorded on subregions of the sagittal hippocampus under the light microscope. The CA1 region was investigated in this research. The white triangle in the left panel marked a pyramidal neuron. **b**–**g** Recordings of postsynaptic currents from acute brain slices of the Sham, the HSP, and the HSPP groups. sEPSCs (**b**–**d**), sIPSCs (**e**–**g**). **b**, **e** Representative traces of sEPSCs, and sIPSCs. **c**, **d, f**, **g** Cumulative distribution plots and bar graphs showed the amplitude (left) and frequency (right) of sEPSCs, and sIPSCs. (For all comparisons, n=9 neurons from 3 rats/group). All data were assessed with One-way ANOVA. Turkey’s multiple comparisons test was performed in comparison of the SHAM *versus* the HSP group, the HSP *versus* the HSPP group and the SHAM *versus* the HSPP group. Data represent means ± SEM. *p<0.05, **p<0.005, ***p<0.001

The impairment of excitatory and inhibitory synaptic transmission in the cortex is often seen as a pathogenic cause of anxiety and depression (Hartmann et al. 2017; Lener et al. 2017). To identify whether hormone withdrawal influenced excitatory and inhibitory neurotransmission, we performed V-clamp recordings on hippocampal CA1 pyramidal neurons from the three model groups. The amplitude and frequency of spontaneous excitatory postsynaptic currents (sEPSCs) in the HSPP group were significantly lower than those in the other two groups (Fig. 2b–d). The HSP group exhibited higher neuron excitability than the other two groups, which was coordinated with the performance in the above behavior tests. Then, we recorded spontaneous inhibitory postsynaptic currents (sIPSCs) in CA1 pyramidal neurons to evaluate whether inhibitory GABAergic inputs were influenced. In the HSPP group, the frequency and amplitude of sIPSCs were considerably higher than those in the HSP group (Fig. 2e–g). Combined with the previous results, the inhibitory inputs of hippocampal CA1 pyramidal neurons of the HSPP group were noticeably larger than the excitatory inputs. These results may suggest that the excitatory and inhibitory neurotransmission of pyramidal neurons in the hippocampal CA1 region in the HSPP group became seriously imbalanced after hormone withdrawal.

### Hormone withdrawal decreased the plasticity of excitatory and inhibitory synapses in the hippocampus

**Fig. 3:**
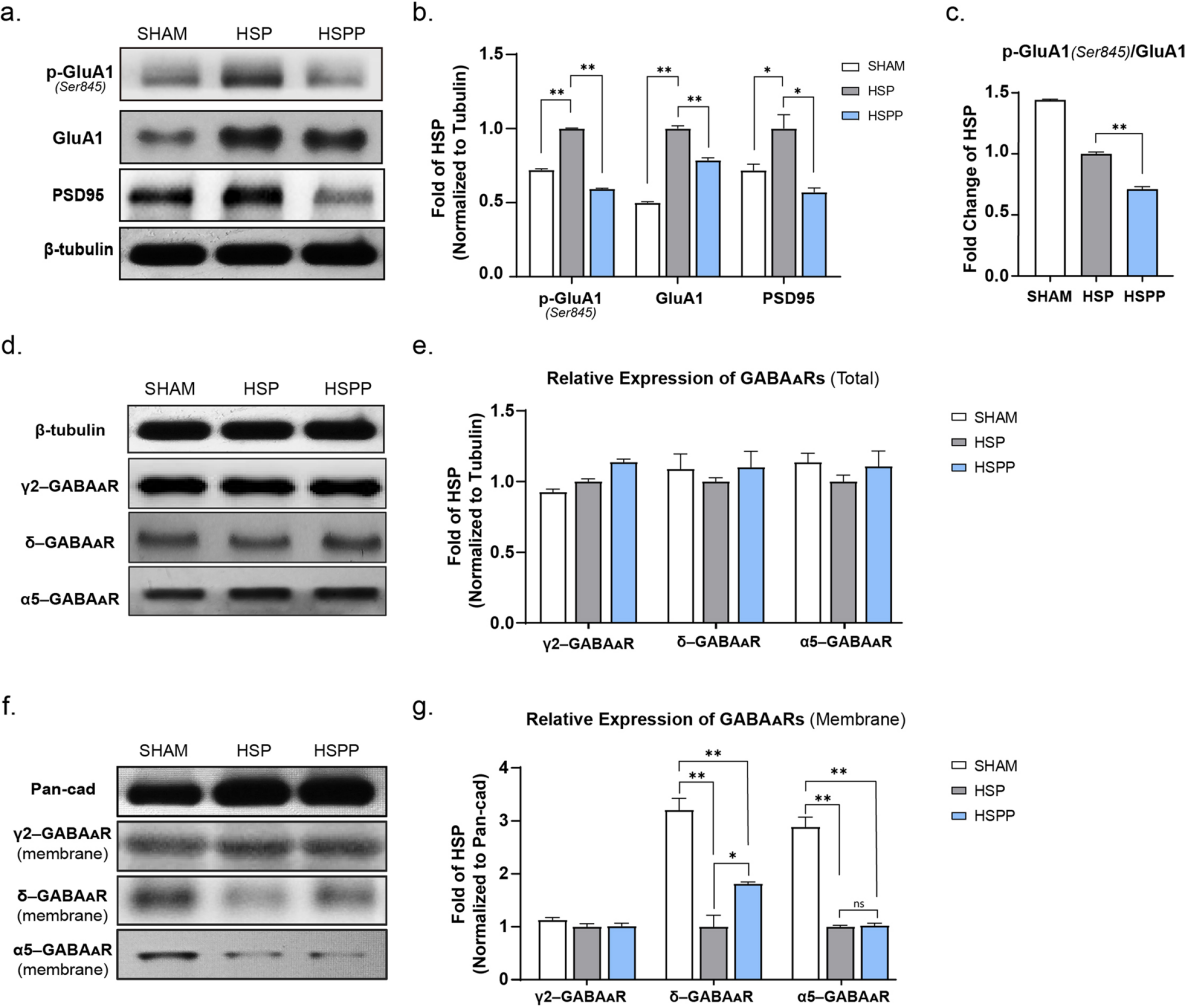
Hormone withdrawal decreased the plasticity of excitatory and inhibitory synapses in the hippocampus. **a**, **b** Relative expression of p-GluA1(Ser845), GluA1, and PSD95 in the hippocampus. **c** The expression of p-GluA1 (Ser845)/GluA1 ratio of three groups. **d**, **e** Relative expression of the γ2, δ, and α5 subunits of GABA_A_Rs in each group in total hippocampal protein level. **f**, **g** Relative expression of the γ2, δ, and α5 subunits of GABA_A_Rs in each group in membrane hippocampal protein level. All the expression of the protein was standardized by the HSP group. All data were assessed with One-way ANOVA. Tukey's multiple comparisons test was performed in comparison of SHAM *versus* the HSPP group. Dunnett's multiple comparisons test was performed in comparison of the HSP *versus* SHAM and the HSP *versus* the HSPP group. Data represent means ± SEM. *p<0.05, **p<0.005, ***p<0.001

To clarify the influences of hormone withdrawal on the structure of synapses, we detected several representative proteins of excitatory and inhibitory synapses, including GluA1, PSD95, and three subunits of GABA_A_Rs (the γ2, δ, and α5 subunits). The δ-subunit has been linked to postpartum depression in previous research (Maguire 2008); the α5-subunit is highly expressed in the hippocampus, mediates extrasynaptic inhibition and is the implicated in depression disorders (Yang et al. 2020); and the γ2-subunit plays a key role in the expression, trafficking and synaptic location of the GABA_A_ heteropentameric receptor in the brain (Pawluski et al. 2015). The levels of GluA1 and p-GluA1 (Ser845) in human brains are commonly used as biomarkers of synaptic plasticity changes (Lopes et al. 2016). As shown in Fig. 3a, b, the expression levels of p-GluA1 (Ser845), GluA1, and PSD95 in the HSPP group were lower than those in the HSP group (Fig. 3a, b). The p-GluA1 (Ser845)/GluA1 ratio was lower in the HSPP group than in the HSP group (Fig. 3c). In evaluating of the expression of inhibitory GABAergic synapse-related proteins, we found that the expression of the γ2, δ, and α5 subunits of GABA_A_Rs in each group demonstrated no difference in total hippocampal protein level (Fig. 3d, e). However, at the membrane protein level, the expression of the δ and α5 subunits was significantly decreased in both the HSP and HSPP groups compared with the sham group (Fig.3f, g). The amounts of δ subunit on the membrane in the HSPP group partly recovered after hormone withdrawal compared with those in the HSP group (Fig. 3g) but were still less than those in the sham-operation group. These results are consistent with the early report that GABA_A_Rs were reduced during pregnancy to coordinate with the high level of hormonal neurosteroids and remained at a low level in postpartum depression patients (Mody 2019). Among the three groups, the expression of the γ2-subunit showed no change either overall or on the membrane (Fig.3d–g). Collectively, these results showed that hormone withdrawal in rats impaired the plasticity of the excitatory and inhibitory GABAergic synapses of the hippocampus, which may cause a disturbance in the excitation and inhibition of CA1 pyramidal neurons.

### Wnt signaling downregulated in the HSPP group

**Fig. 4:**
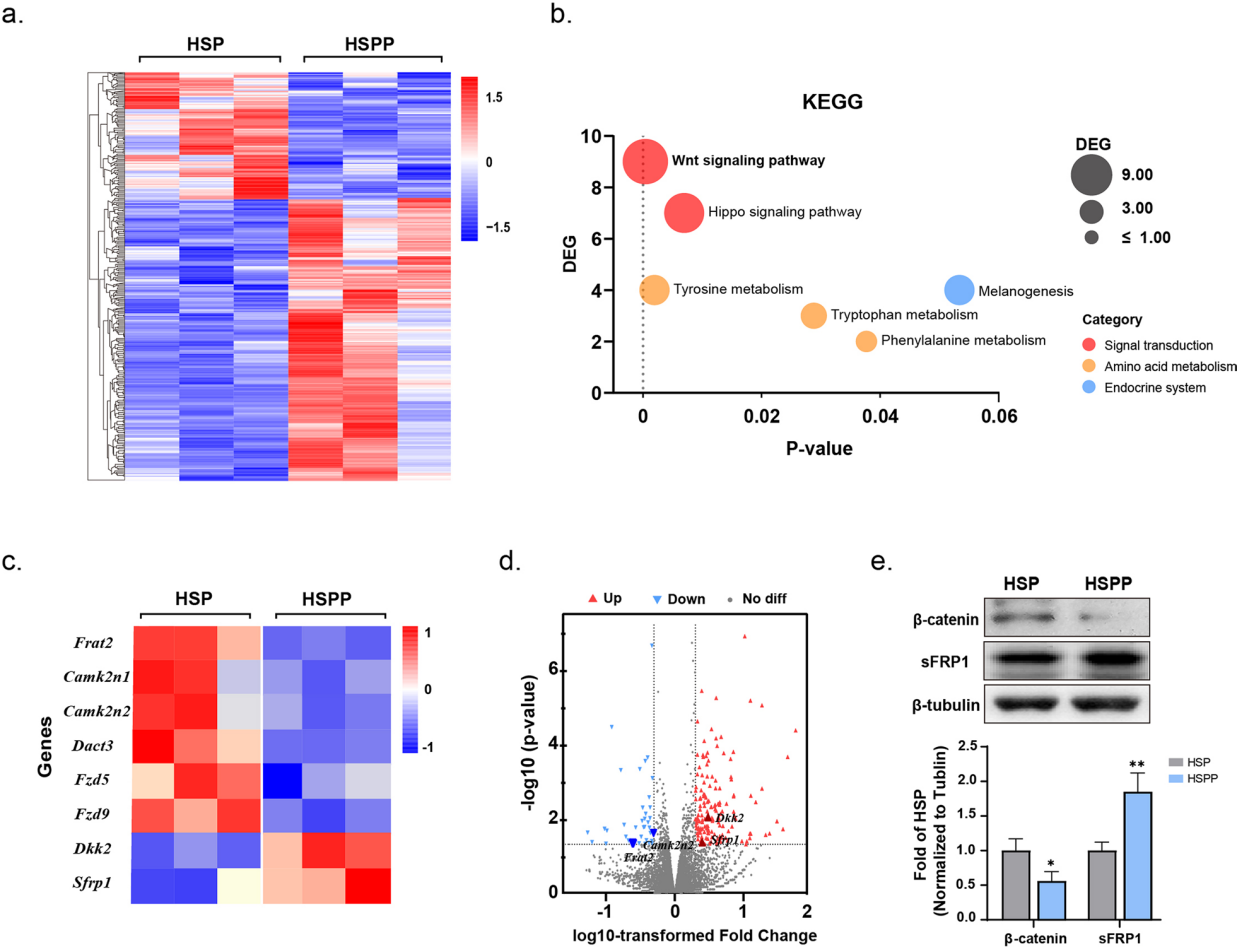
Wnt signalling downregulated in the HSPP group. Gene expression profile analysis of the hippocampus of the HSP and HSPP groups. **a** Differentially expressed genes (DEGs) between the HSP and HSPP groups, with 215 upregulated (red) and 97 downregulated (blue). (Using |log2-fold change|>0.584 (1.5-fold change) and p=0.05 as inclusion criteria). **b** The KEGG enrichment analysis of the HSP and HSPP groups. The Wnt signalling pathway and several amino acid metabolic pathways associated with depression were enriched. **c**, **d** A heatmap and a volcano plot of several key genes involved in Wnt signalling of the HSP and HSPP groups. **e** Relative expression of β-catenin and sFRP1 (secreted frizzled protein), standardized by the HSP group. These data were assessed with unpaired t-tests. Data represent means ± SEM. *p<0.05, **p<0.005

To explore the underlying mechanism of the depressive-like phenotypes induced by reproductive hormone withdrawal, we performed RNA-seq on hippocampal tissue from the HSP and HSPP groups. Gene expression profile analysis revealed that hormone withdrawal induced dynamic changes in the gene transcriptome. Using |log2-fold change|>0.584 (1.5-fold change) and p=0.05 as inclusion criteria, a total of 312 differentially expressed genes (DEGs) were screened between the two groups, with 215 upregulated and 97 downregulated (Fig. 4a). The DEG biological pathways and functions were then analysed using Kyoto Encyclopedia of Genes and Genomes (KEGG) pathway analysis. The Wnt signaling pathway was identified to be significantly enriched in the KEGG enrichment analysis (Fig. 4b). Several amino acid metabolic pathways associated with affective disorders were also enriched (Fig. 4b). A heatmap and a volcano plot were utilized to display several key genes involved in Wnt signaling (Fig. 4c, d).

To verify whether the Wnt signaling pathway was blunted in the HSPP group, we detected the expression level of Wnt signaling-related proteins in the hippocampus. The level of β-catenin, a key mediator of the canonical Wnt signaling pathway (a.k.a. Wnt/β-catenin signaling) that contributes to the pathophysiology of depressive disorders (Gao et al. 2021), was decreased in the HSPP group (Fig. 4e). sFRP1 (secreted frizzled protein), which can inhibit both canonical and noncanonical Wnt pathways and is reportedly linked to neurogenesis processes and depression disorders (Seib et al. 2013), was markedly higher in the HSPP group (Fig. 4e). In conclusion, after hormone withdrawal, Wnt signaling was blunted in the HSPP group.

### Activation of Wnt signaling increased excitatory and inhibitory synaptic transmission in the hippocampus

**Fig. 5:**
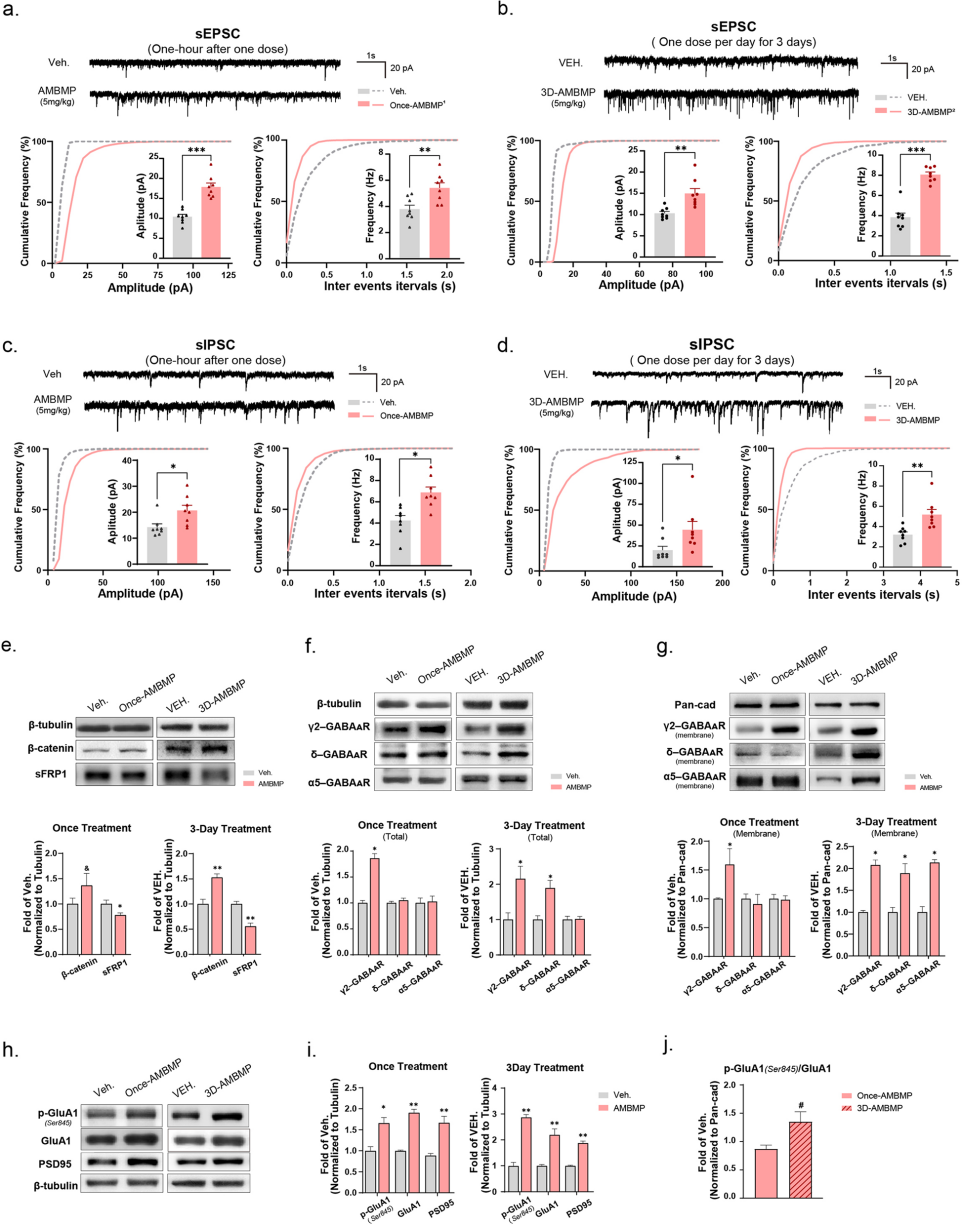
Activation of Wnt signalling increased excitatory and inhibitory synaptic transmission in the hippocampus.** a**–**d** Recordings of postsynaptic currents of CA1 pyramidal neurons from acute brain slices of the vehicle and AMBMP-treatment groups. Representative traces (up) and cumulative distribution plots and bar graphs showed the amplitude (left) and frequency (right) of sEPSCs, and sIPSCs. **a**, **c** Recorded one hour after one treatment regimen with AMBMP (i.p. 5mg/kg). **b**, **d** Recorded one day after the 3-day treatment regimen of AMBMP (i.p. one dose of 5mg/kg AMBMP per day for 3 continuous days). For all comparisons, n=8 neurons from 3 rats/group. **e** Relative expression of β-catenin and sFRP1 on hippocampus from one treatment and 3-day treatment regimens. **f**, **g** Relative expression of the γ2, δ, and α5 subunits of GABA_A_Rs in total and membrane hippocampal protein level in two treatment regimens. All the expression of the protein was standardized by the vehicle group of each treatment. **h**, **i** Relative expression of p-GluA1(Ser845), GluA1, and PSD95 in the hippocampus in two treatment regimens. An unpaired t-test was performed to assess the expression differences between the vehicle and AMBMP-administrated groups. Data represent means ± SEM. *p<0.05, **p<0.005, ***p<0.001, &p=0.07 **j** The expression of p-GluA1 (Ser845)/GluA1 ratio in two treatment regimens. Unpaired t-test test was performed in comparison of once-AMBMP *versus* the 3D-AMBMP group. The data were normalized to the vehicle group of each regimen. Data represent means ± SEM. #p=0.06. ^1^once-AMBMP: sampled one hour after one treatment (i.p. AMBMP, 5mg/kg)**.** ^2^3D-AMBMP: sampled one day after 3-day treatment (i.p. AMBMP, 5mg/kg per day for 3 continuous days)

To verify whether the activation of Wnt signaling has a regulatory impact on synaptic transmission, we administered a Wnt signaling agonist AMBMP to 2 to 3-week-old female SD rats and assessed the function of synapses by analysing synaptic-related protein levels in the hippocampus and recording sIPSCs and sEPSCs in pyramidal neurons from the CA1 region. Two strategies for drug administration were designed to find the most effective one for the following experiment: intraperitoneal injected (i.p.) of 5mg/kg AMBMP one or one i.p. dose of 5mg/kg AMBMP per day for 3 continuous days. The functional analyses were performed one hour or one day later for the once or three-continuous-day treatment groups. The results showed that the amplitude and frequency of sEPSCs and sIPSCs were both increased after one or 3 continuous days (3-day in below) of AMBMP administration (Fig. 5a–d). Next, protein expression studies were conducted on identical rats’ other side of the hippocampus, of which one side of hippocampus had been prepared for brain slices for recordings. After once and 3-day treatment, the expression level of β-catenin increased noticeably, and the level of sFRP1 was reduced, indicating that the Wnt signaling pathway had indeed been activated (Fig. 5e). In the analysis of GABA_A_R subunits expression, once treatment had no significant effect on upregulating GABA_A_Rs except the γ2 subunit (Fig. 5f-g). However, the expression of γ2-GABA_A_Rs and δ-GABA_A_Rs markedly increased at both the total and membrane protein levels in the 3-day treatment group (Fig. 5f, g). GABA_A_Rs containing α5-subunits increased considerably on neuron membranes in the 3-day administration group as well (Fig. 5g). In addition, in both treatment groups, p-GluA1(Ser845) and GluA1 in the neuronal membrane and PSD95 were found to be expressed more strongly in the hippocampus. Moreover, the p-GluA1 (Ser845)/GluA1 ratio was significantly increased in the 3-day administration group (Fig. 5h–j). In conclusion, the activation of Wnt signaling by administering AMBMP upregulated neuronal excitability and had a substantial effect after once treatment. Nevertheless, the three-day administration regimen was more efficient at upregulating functional GABA_A_Rs at the protein level in addition to regulating neuronal activity. Therefore, we chose the 3-day treatment for further research on the HSP/HSPP models.

### Reactivation of Wnt signaling in the HSPP group alleviated the anhedonic behaviors and abnormal synaptic transmission induced by hormone withdrawal

**Fig. 6:**
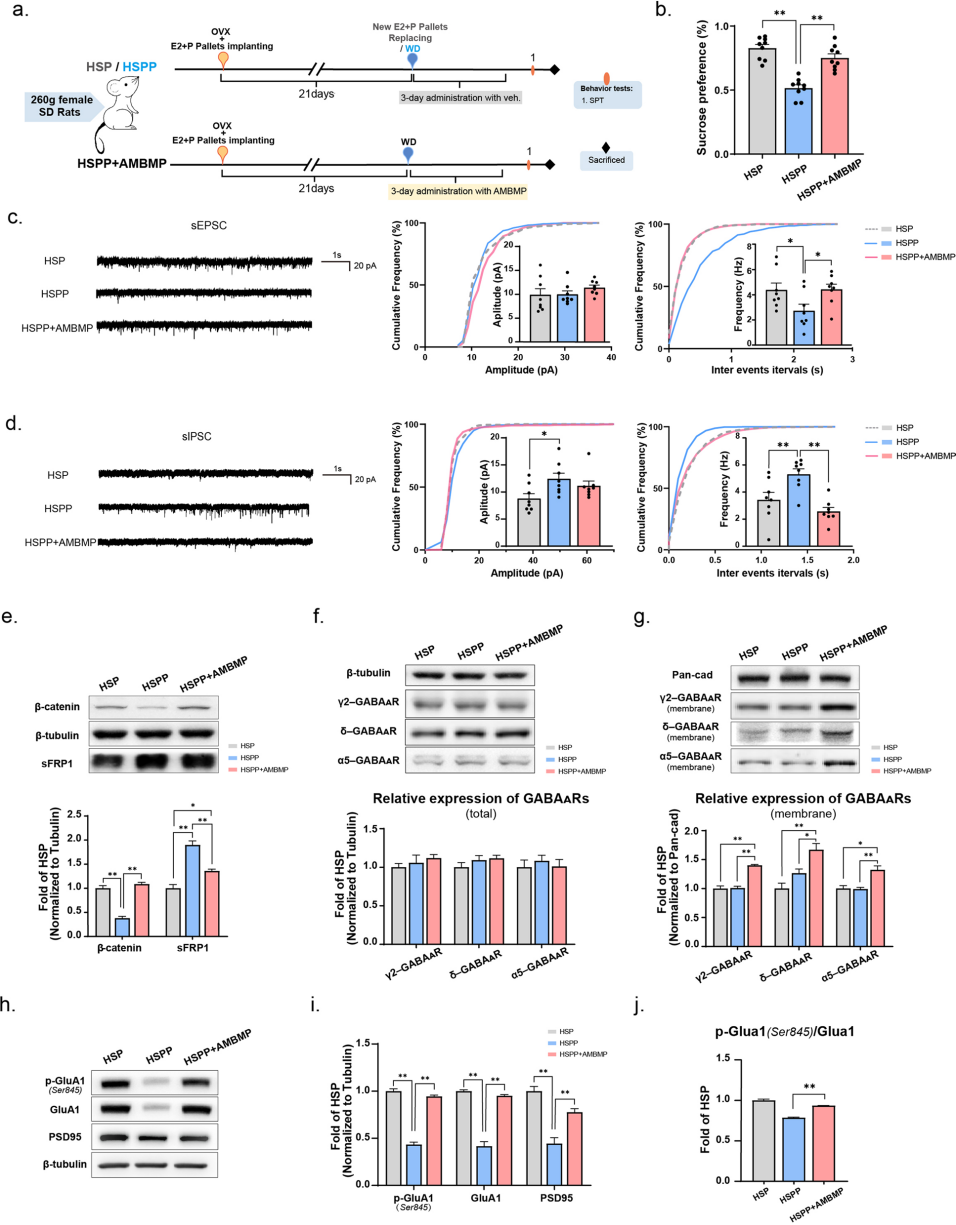
Reactivation of Wnt signalling in the HSPP group alleviated the anhedonic behaviour and abnormal synaptic transmission induced by hormone withdrawal.** a** The schedule for administration with AMBMP on the HSPP model and the arrangement behavior tests. The HSP and HSPP group were injected with the same volume of a vehicle solvent with the AMBMP working solution to set as control. SPT test was performed after the 3-day administration of AMBMP. (HSP, n=9; HSPP, n=9; HSPP+AMBMP, n=9). **b** The sucrose preference test (SPT) was measured for two hours in the three treatment groups. **c**, **d** Recordings of sEPSCs and sIPSCs from acute brain slices of the HSP, HSPP, and HSPP+AMBMP groups. Representative traces (left), cumulative distribution plots, and bar graphs showed the amplitude (left) and frequency (right) of sEPSCs, and sIPSCs. (For all comparisons, n=8 neurons from 3 rats/group). **e** Relative expression of β-catenin and sFRP1 on hippocampus from three model groups with the 3-day treatment. **f**, **g** Relative expression of the γ2, δ, and α5 subunits of GABA_A_Rs in total and membrane hippocampal protein level in 3 groups. **h**, **i** Relative expression of p-GluA1(Ser845), GluA1, and PSD95 in the hippocampus in three treatment groups. **j** The expression of p-GluA1 (Ser845)/GluA1 ratio in two treatment regimens. All the expression of the protein was standardized by the HSP group. All data were assessed with One-way ANOVA. Tukey's multiple comparisons test was performed in comparison of the HSP *versus* the HSPP group, HSP *versus* the HSPP+AMBMP group, and the HSPP *versus* the HSPP+AMBMP group. Only significant comparisons were displayed in the figures. Data represent means ± SEM. *p<0.05, **p<0.005

We then investigated whether restoring the blunted Wnt signaling of the HSPP group could alleviate the anomalous changes induced by hormone withdrawal. Right after the withdrawal procedures, female adult rats in the HSPP+AMBMP group were pretreated with AMBMP for 3 days (5mg/kg, one dose per day, i.p.) before the behaviors test (Fig. 6a). The sucrose preference percentage significantly increased after AMBMP administration in the HSPP+AMBMP group, indicating a reversion of the anhedonia induced by hormone withdrawal by Wnt signaling agonism (Fig. 6b). As shown in Fig. 6a, the same batch of rats was sacrificed after the sucrose preference test for synaptic-related functional analysis. Compatible with the SPT result, whole cell patch-clamp recording of pyramidal neurons in the CA1 hippocampus showed abnormal decreases in the frequency of sEPSCs and increases in the frequency of sIPSCs that were all reversed by AMBMP administration (Fig. 6c, d). The upregulation of β-catenin expression and the downregulation of sFRP1 expression in the HSPP+AMBMP group in the western blotting analysis indicated that AMBMP did work to restore Wnt signaling (Fig. 6e). The detection of synaptic-related proteins showed that the expression of γ2-GABA_A_Rs in the hippocampus increased at both the total and membrane levels in the HSPP+AMBMP group (Fig. 6f, g). GABA_A_Rs including the δ- and α5-subunits, increased markedly at the membrane level after AMBMP administration (Fig. 6g**)**, which was similar to the results in Fig. 5f, g) The expression of p-GluA1 (Ser845), GluA1, and PSD95 was recovered by AMBMP administration in the HSPP+AMBMP group (Fig. 6h, i) as was the p-GluA1 (Ser845)/GluA1 ratio (Fig. 6j). These results suggest that AMBMP administration could also improve the plasticity of synapses. In summary, the results of our analyses of behaviors, electrophysiology, and synapse-related protein expression suggested that the reactivation of Wnt signaling alleviated the anhedonic behaviors and abnormal synaptic transmission induced by hormone withdrawal.

## Discussion

This research reveals the molecular mechanism of hopelessness and anhedonia in a hormone-simulated postpartum period (HSPP) rat model, that is, the disturbance of excitatory and inhibitory CA1 pyramidal neurons in the hippocampus induced by drastic hormonal fluctuations. And this disturbance was due to glutamatergic and GABAergic synaptic plasticity dysfunction. This result might suggest the possibility that impaired E-I balance may play an important role in the HSPP model. The findings of this research also complement those of previous studies on the GABA_A_Rs dysfunction involved in PPD: malfunction of canonical Wnt signaling in the HSPP group might be one of the underlying molecular mechanisms of GABA_A_Rs recovery dysfunction, which caused synaptic transmission dysfunction. Reactivating Wnt signaling in the perinatal period might alleviate the abnormal synaptic activity and adverse mental symptoms of individuals with PPD.

Variations in reproductive hormone levels represent an obvious risk factor for postpartum depression (Payne and Maguire 2019). Oestrogen and progesterone have been established to influence neuroplasticity in many brain regions, particularly mediating neurogenesis in hippocampal formation (Sheppard et al. 2019; Uzair et al. 2020). We confirmed the familiar consequences identified in previous research (Suda et al. 2008) that animals displayed hopelessness and anhedonia in the escape failure test after inescapable stress and the sucrose preference test after hormone withdrawal (Fig. 1b–d). In the open field test, the percentage of central distance/total distance significantly decreased in the HSPP group, indicating that the rats also had symptoms of anxiety (Fig. 1f). Anxiety and depression are usually reported concurrently in the postpartum stage in humans (Cantwell 2021). Moreover, we demonstrated the electrophysiological responses of animals’ CA1 pyramidal neurons at different hormone levels. This might reveal the potential molecular mechanism through which hormones trigger depression-related behaviors.

Interestingly, we found that body weight significantly decreased in the HSPP group after rats were stressed with an inescapable electric shock (Additional file 1: Fig. S1a, b). Body weight gain was remarkably influenced by hormone levels. The body weights of the HSP and HSPP groups in the simulated pseudopregnancy period were remarkably lower than those of the sham-operation group (Additional file 1: Fig. S1a). However, upon hormone withdrawal, the body weight of the rats in the HSPP group increased distinctly compared to that of the rats in the HSP group on PD2 (Additional file 1: Fig. S1b). Furthermore, the body weights of the HSPP group tended to decrease quickly after an unavoidable electric shock on PD4, this body weight loss was noticeable in comparison to the other two groups on PD5 (Additional file 1: Fig. S1b). On PD6, the body weight of the HSPP group again increased and it was significantly greater than that of rats in the HSP group. (Additional file 1: Fig. S1). These results provide evidence that hormone withdrawal may induce susceptibility to stress in females. Eating disorders such as anorexia, malnourishment, or abnormal eating attitudes and behaviors are common in mental disorders, as well as in postpartum depression disorder. Stress-susceptible mice, which show a depressive-like phenotype, display significant changes in body weight and metabolic disturbances. Obesity in the postgestation and postpartum periods is related to maternal affective symptoms and baby-caring behaviors (Cantwell 2021; Lutter et al. 2008; Faria-Schutzer et al. 2018). Considering that the inescapable foot shock was strong stress that might be an additional factor that could induce depressive behaviors, related electrophysiology responses, and variations in synaptic plasticity (Baka et al. 2017; Dygalo et al. 2020; Yang et al. 2020; Pawluski et al. 2015), we did not perform the escape failure test in subsequent behaviors experiments to ensure the results in later experiments were mediated only by variable hormone levels and wnt agonist (AMBMP) treatment. Nonetheless, the anhedonia shown in the SPT was a core and persuasive indicator of depression (Liu et al. 2018).

The plasticity and homeostasis of GABA_A_Rs have been implicated in the pathology of PPD, especially the δ-subunit, which is mainly found in extrasynaptic GABA_A_Rs and is the target of neurosteroids (Maguire 2008). The δ- and α5-subunit of GABA_A_Rs were observed to be significantly influenced by hormone fluctuations in this work, the α5-subunit, which is highly expressed in the hippocampus and reported related to depressive disorders, and modulated tonic inhibition in extra-synapses (Mohamad and Has 2019). These two subunits decreased remarkably while hormone levels were maintained at the pregnancy level in this work. However, another important subunit of GABA_A_Rs, the γ2-subunit, showed no change with altered hormone levels. Based on these changes in GABA_A_R subunits, we propose a possible explanation for the diverse E/I ratio recorded in the Sham, HSP, and HSPP brain slices. Since the metabolites of ovarian hormones could positively allosteric modulate GABA_A_Rs (Maguire 2019), the presynaptic and postsynaptic responses to variable levels of neuroactive steroids resulted in various frequencies of vesicular GABA release and diverse amounts or functions of GABA_A_Rs (Herd et al. 2007). Animals of the SHAM group were regarded as being in the oestrous or diestrous cycle, in which reproductive hormones fluctuated in a small range. The electrophysiological responses in this group demonstrated the basic excitability of the rats (Fig. 2). In the HSP group, however, the high concentration of neurosteroids made the functional GABA_A_Rs robust in both presynapses and postsynapses, and the GABA_A_Rs adaptive decreased, so the excitability of GABAergic neurons increased, and the amplitude of sIPSCs recorded in pyramidal neurons was thus less than that in the SHAM group. The higher frequency (the shorter intervals) of the HSP group was due to the augmented GABA_A_Rs resulting from high levels of neuroactive steroids. The amounts of δ-subunits displayed a trend in the recovery of GABA_A_Rs in the HSPP group (Fig. 3g), which might be the reason that the recorded amplitude of sIPSCs in this group was larger than that in the HSP group. A previous study reported that neurosteroids prolong the decay of sIPSCs and mIPSCs (Cooper et al. 1999). This promotes a prolonged open state of GABA_A_Rs might disappear sensitively after hormone withdrawal; hence, the frequency of sIPSCs was higher in the HSPP group. Furthermore, the levels of GluA1 and p-GluA1 (Ser845) in the hippocampus were significantly higher in the HSP group, as was the level of PSD95 (Fig. 3a, b), indicating the higher plasticity of the excitatory synapses in this group. The p-GluA1 (Ser845)/GluA1 ratio of the HSPP group was significantly lower (Fig. 3c), also demonstrating the lower synaptic plasticity of the group (Lopes et al. 2016; Yao et al. 2004). In EPSC recordings of brain slices, the HSPP group displayed the lowest excitability among the three groups. Combined with the features of IPSCs mentioned above, the HSPP group demonstrated an extreme imbalance of excitation and inhibition (Fig. 2), which might be the underlying mechanism of depressive-like behaviors. Indeed, the electrophysiological characteristics of the HSP group revealed that the animals in this group were more excited, with a significantly higher E/I ratio than the other two groups (Fig. 2f). These electrophysiological performances matched the results of the behaviorsal experiments. Animals in the HSP group barely had any escape failures, and their escape latency was significantly lower than that of the Sham group (Fig. 1b, c). However, the reason that the p-GluA1 (Ser845)/GluA1 ratio in the Sham group was higher than that in the HSP group is unknown and needs to be further investigated. Moreover, further investigations are needed to determine how other types of neurons, such as inhibitory interneurons, respond to neuroactive steroids and their interactions with pyramidal neurons.

Oestrogen and progesterone have been reported to mediate Wnt signaling (Fortress and Frick 2016). Therefore, we speculate that the dysfunction of Wnt signaling in the HSPP group resulted from the variation in hormone levels. In the RNA-seq experiment, genes related to canonical and noncanonical Wnt signaling were found to be changed. Overall Wnt signaling was blunted in the HSPP group at the mRNA level (Fig. 4c, d). Previous research has reported that activating Wnt signaling in the canonical and noncanonical pathways with multiple Wnt ligands could upregulate GABA_A_Rs (Cuitino et al. 2010). In this study, however, we detected changes only in canonical Wnt signaling (a.k.a. Wnt/β-catenin signaling) in the western blotting analyses. The expression of β-catenin, a key mediator of the canonical Wnt signaling pathway, the decreased of which contributes to the pathophysiology of depressive disorders (Gao et al. 2021), was significantly decreased in the hippocampus of the HSPP group (Fig. 4e). Therefore, we chose to interfere with Wnt signaling with an agonist of canonical Wnt signaling (AMBMP) in the HSPP rat model for further investigation.

We verified that the fluctuation of hormones mainly influenced the amounts of GABA_A_Rs on the neuron membrane (Fig. 3d–g). GABA_A_Rs transport to the membrane is a quick process, as a previous study found that it took only 15 min to increase the expression of GABA_A_Rs by activating Wnt signaling via a wnt ligand (Cuitino et al. 2010). Therefore, we prepared rapid brain slices one hour after intraperitoneal injection of AMBMP to record the IPSCs and EPSCs. The other side of the hippocampus of the same rat was used to analyse protein expression. We wanted to verify whether activating Wnt signaling with AMBMP could modulate electrophysiological activities and protein expression quickly. Moreover, distinct depressive-like behaviors were aroused three or four days after hormone withdrawal in previous research (Suda et al. 2008). Therefore, we also employed three days of AMBMP treatment and then examined the electrophysiological and protein changes. One-hour after once administration was found to rapidly modulate the function of CA1 pyramidal neurons by enhancing excitatory and inhibitory currents simultaneously (Fig. 5a, c). After 3 days of continuous administration, much more significant changes were observed at the protein level: the expression of GABA_A_Rs, GluA1, p-GluA1, PSD95, and the p-GluA1 (Ser845)/GluA1 ratio were all increased (Fig. 5e–j), suggesting that the function and plasticity of excitatory and inhibitory synapses were both enhanced by Wnt signaling activation. Therefore, we chose to perform the 3-day treatment on the HSPP model for subsequent tests. We wanted to see whether continually administering AMBMP for 3 days to activate Wnt signaling could alleviate the depressive-like behaviors and the disturbance of the E/I balance that manifestly occurred on the 4th day after hormone withdrawal. However, we noticed that the γ2, δ, and α5-GABA_A_Rs subunits increased after Wnt signaling was activated (Fig. 5f, g). Notwithstanding, even though Wnt signaling was confirmed to be downregulated in the HSPP model (Fig. 4c–e), only the δ and α5 subunits displayed significantly altered expression (Fig. 3f, g). We speculated that other signaling pathways may be involved in the regulation of GABA_A_R functions, which needs to be investigated further.

The 3-day treatment of the HSPP group with AMBMP led to effects such as an increased sucrose preference (Fig. 6b). Combined with the restoration of the E/I ratio in the brain slices after administration (Fig. 6c–e), we speculated that reactivated Wnt signaling alleviated depressive-like behaviors in the hormones-simulated postpartum period (HSPP) rat model. Brezanolone, a kind of neuroactive steroid drug that targets GABA_A_Rs, was administered in the clinic to PPD patients (Meltzer-Brody et al. 2018). Neuroactive steroids used in PPD treatment equivalently prolonged the mitigation period of GABA_A_R dynamic changes with hormone levels. Nevertheless, activating Wnt signaling not only provided an alternative strategy to augment GABAergic transmission to rebalance the E/I imbalance of neurons but also enhanced the E/I input and neuronal activity in the hippocampus by increasing glutamate transmission and promoting the plasticity of synapses (Fig. 6h–j). However, further investigation is needed to find a more selective way to activate Wnt signaling that regulates only extrasynaptic GABA_A_Rs, which are mainly influenced by hormones. Therefore, this treatment might be more effective.

There were some limitations in this research. Western blotting and RNA-seq analyses were performed on the entire hippocampus. The results will be more valuable and convincing when examined on a specific brain region such as quantifying immunostaining images of CA1 pyramidal neurons or even on specific types of neurons such as GABAergic neurons. Moreover, it should be noted that this study discussed only one inducement of postpartum depression: hormonal fluctuations. The plasticity and neuronal activities of the hippocampus in the HSPP rat model were the focus of this work. Indeed, multiple signaling pathways and systems have been implicated in the neurobiology of PPD. Numerous factors can cause PPD in adult women, including genetic factors (Roy et al. 2020) and social factors such as low social support, marital difficulties, and negative life events(Stewart and Vigod 2016).

The findings presented here shed new light on the potential mechanism underlying the difficulty in restoring GABA_A_R quantity in postpartum depression patients. Activating Wnt signaling could be a prospective therapeutic regimen and a supplement to neuroactive steroid intervention for women suffering from postpartum depression. We hope that this study will provide some assistance and support for basic research on postpartum depression treatment options.

## Conclusions

Overall, this research revealed that the blunted Wnt signaling might contribute to the molecular mechanism of how hormone withdrawal induced depressive-like behaviors, disturbing the balance of excitatory and inhibitory transmission in the hippocampus. Administrated with the canonical Wnt signaling agonist to restore Wnt signaling remediated depression-related anhedonia symptoms and coordinated the excitation/inhibition ratio by collectively enhancing the plasticity of GABAergic and glutamatergic synapses. These findings could provide a potential target and a complementary treatment strategy for postpartum depression.

## Supplementary Information

**Additional file 1.** **Figure S1.** Change of body weight in three model groups. **a** Changes in body weight in the three model groups. The first measurement was taken on GD0 (the day performed OVX or sham operations). During the gestation period, body weights were measured on GD4 (Day 4 of the gestation period), GD8, GD12, GD16, and GD21. During the postpartum period, body weight was measured on PD2 (Day 2 of the postpartum period), PD4, and PD6. The red arrow indicated the day to procedure hormone withdrawal (on GD21). The yellow arrow indicated the day proceeded LH stress (on PD4). **b** The gain in the body weight of each group on each test day. (HSP, n = 10; HSPP, n = 10; HSPP + AMBMP, n = 10). Data on each test day were assessed with One-way ANOVA. Tukey's multiple comparisons test was performed in comparison of the HSP versus the HSPP group. Data represent means ± SEM. *p < 0.05. ^1^Hor. WD: hormone withdrawal. ^2^LH-stress: learned-helpless stress (inescapable electric foot shock).

**Additional file 2.** **Supplementary table 1-3:** The formulations of Artificial Cerebrospinal Fluid (aCSF).

## Data Availability

Data sharing not applicable to this article as no data-sets were generated or analyzed during the current study.

## Abbreviations

PPD: Postpartum depression
HSP: Hormones-simulated pseudopregnancy
HSPP: Hormones-simulated postpartum period
AMPA: α-Amino-3-hydroxy-5-methyl-4-isoxazolepropionic acid
GABAARs: γ-Aminobutyric acid sub-type A receptors
E/I: Excitation/inhibition
WD: Withdrawal
OVX: Ovariectomized
GD: Gestation period
PD: Postpartum period
OFT: The open field test
FR: Fixed-ratio
SPT: The sucrose preference test
AMBMP: 2-Amino-4-[3,4-(methylenedioxy)benzylamino]-6-(3-methoxyphenyl)pyrimidine
TCF: Transcription factor
BW: Body weight
NS: Normal saline
i.p.: Intraperitoneally injected
aCSF: Artificial cerebrospinal fluid
sIPSC: Spontaneous inhibitory postsynaptic current
sEPSC: Spontaneous excitatory postsynaptic current
