# Supplementary figures and images for: Restoring Wnt signaling in a hormone-simulated postpartum depression model remediated imbalanced neurotransmission and depressive-like behaviors
Source: Mol Med. 2023 Jul 25;29:101. doi: 10.1186/s10020-023-00697-4 (PMC10369844; doi:10.1186/s10020-023-00697-4)

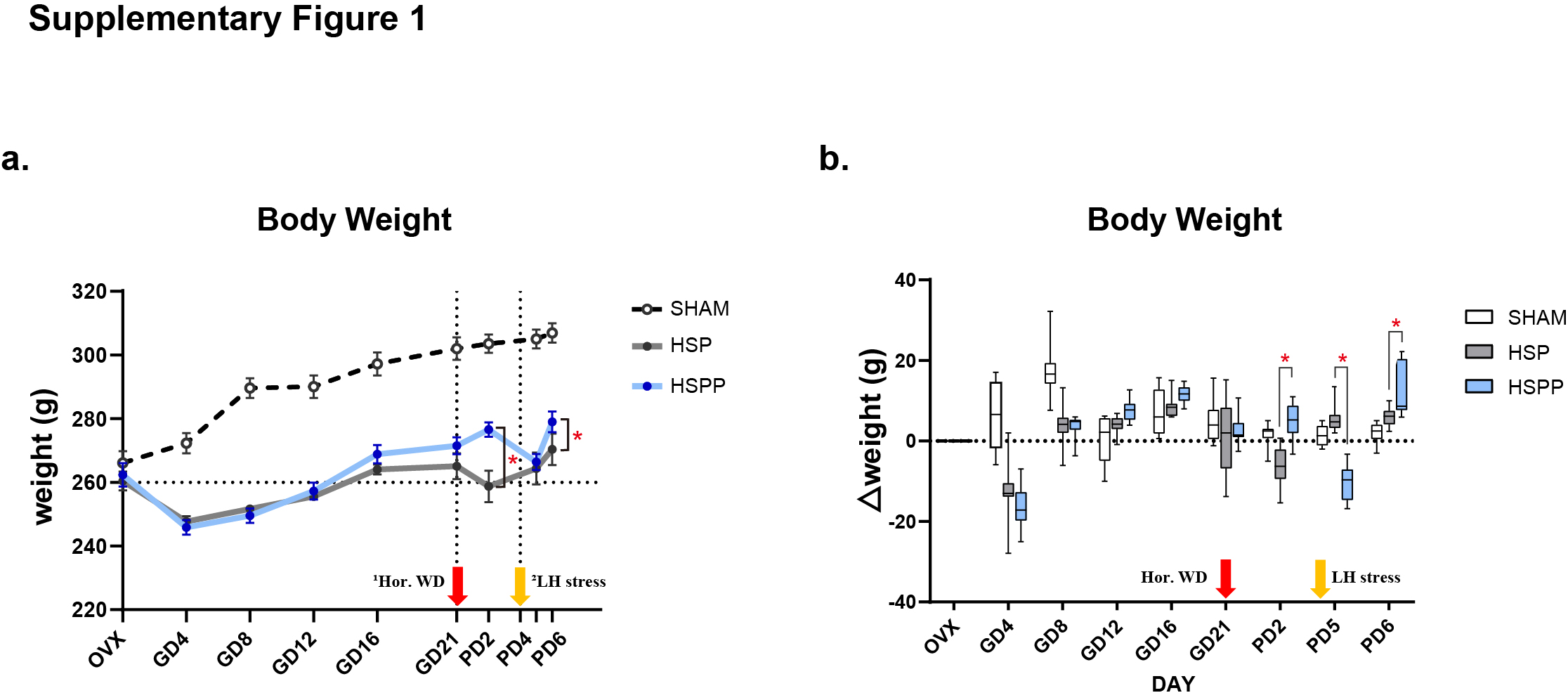

Supplement: Supplementary file 1 — Additional file 1. Figure S1. Change of body weight in three model groups. a Changes in body weight in the three model groups. The first measurement was taken on GD0 (the day performed OVX or sham operations). During the gestation period, body weights were measured on GD4 (Day 4 of the gestation period), GD8, GD12, GD16, and GD21. During the postpartum period, body weight was measured on PD2 (Day 2 of the postpartum period), PD4, and PD6. The red arrow indicated the day to procedure hormone withdrawal (on GD21). The yellow arrow indicated the day proceeded LH stress (on PD4). b The gain in the body weight of each group on each test day. (HSP, n = 10; HSPP, n = 10; HSPP + AMBMP, n = 10). Data on each test day were assessed with One-way ANOVA. Tukey's multiple comparisons test was performed in comparison of the HSP versus the HSPP group. Data represent means ± SEM. *p < 0.05. 1Hor. WD: hormone withdrawal. 2LH-stress: learned-helpless stress (inescapable electric foot shock). [file 10020_2023_697_MOESM1_ESM.jpg]
